# First person – De-Li Xu

**DOI:** 10.1242/bio.039685

**Published:** 2018-12-15

## Abstract

First person is a series of interviews with the first authors of a selection of papers published in Biology Open, helping early-career researchers promote themselves alongside their papers. De-Li Xu is first author on ‘Seasonal variations in cellular and humoral immunity in male striped hamsters (*Cricetulus barabensis*)’, published in BiO. De-Li conducted the research described in this article while a PhD student in Dehua Wang's lab at the Institute of Zoology, Chinese Academy of Sciences. He is now a doctor, investigating ecological immunology in small mammals.


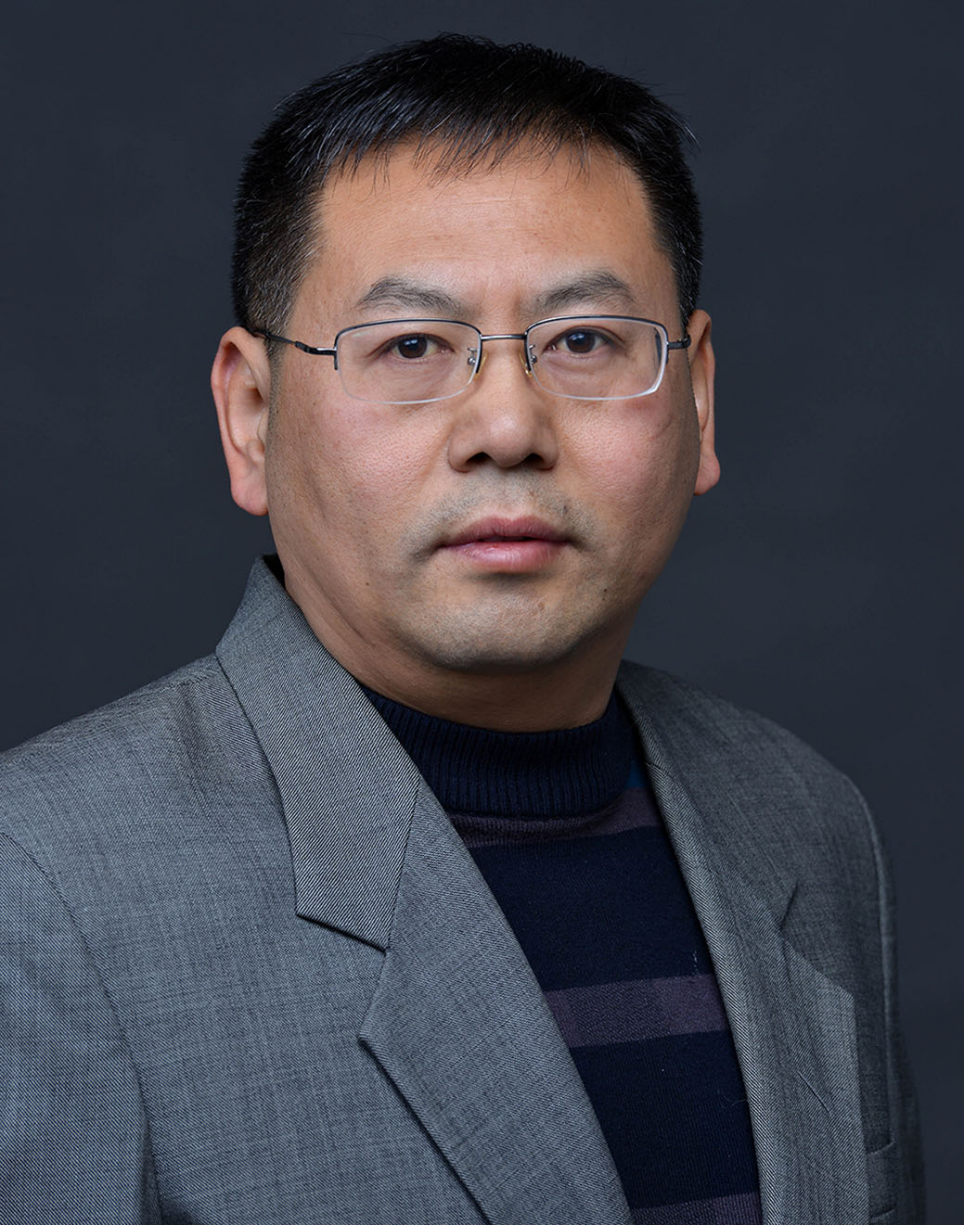


**De-Li Xu**

**What is your scientific background and the general focus of your lab?**

My academic training is in physiological ecology and biochemistry, and my research focuses on ecoimmunology. In other words, we are more interested in why immune responses vary in wild animals and the possible consequences of the changed immunity.

**How would you explain the main findings of your paper to non-scientific family and friends?**

Immune function plays an important role in protecting humans and animals against environmental pathogens such as viruses and bacteria. Their immunity usually shows seasonal changes, which is crucial for them to adapt to different seasonal environments. We found that cellular immunity was lowest in summer in striped hamsters, indicating that their ability to fight off intracellular pathogens (i.e. virus) was lowest in summer. However, humoral immunity was lowest in the fall, implying that hamsters had the lowest capacity to fight against extracellular pathogens, such as bacteria, in this season. These results indicate that survival capacity might change seasonally in hamsters.

**What are the potential implications of these results for your field of research?**

Different components of the immune system showed different seasonal variations, implying that hamsters might utilize different immune responses to deal with different pathogens during the four seasons.

**What has surprised you the most while conducting your research?**

We were surprised to find the survival capacity in hamsters showed seasonal changes. After finishing the research, I found that many new questions arose and these questions are also worth further investigation. The nature of research is never-ending.

“The nature of research is never-ending.”

**What, in your opinion, are some of the greatest achievements in your field and how has this influenced your research?**

Research on the effect of ecological factors on immune function have been carried out in a variety of wild animals, and the mechanisms underlying the changes in immunity have also received much attention. These achievements help us to focus on important unanswered questions in ecological immunology.

**What do you think could improve the professional research of early-career scientists?**

Firstly, it is important for us to track the recent advancements in the field of ecoimmunology. Secondly, it is crucial for us to do research step-by-step and to focus on interesting and important scientific questions. Finally, it is key to adopt integrative methods including classic and new technologies to deal with these questions.

**Figure:**
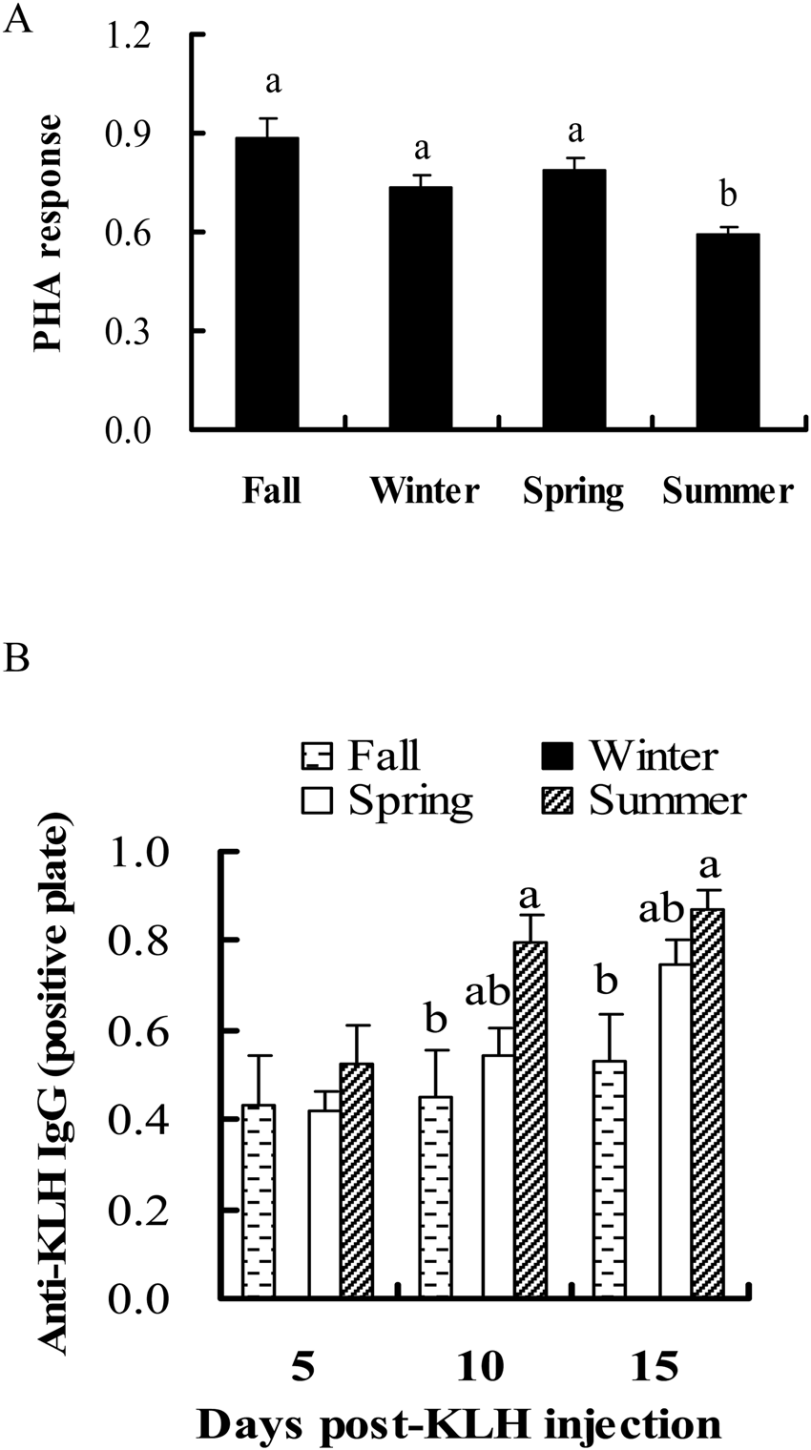
**Animal Physiological Ecology.** Seasonal changes of cellular (A) and humoral immunity (B) in male striped hamsters. Different letters above the column indicate significant differences at *P*<0.05.

**What's next for you?**

Research in animal physiological ecology and ecoimmunology attract me, and I will continue with research in these fields. I would also like to try and answer new questions that arise from this research. Investigating unknown questions and obtaining their answers is great fun to me.

“Investigating unknown questions and obtaining their answers is great fun to me.”
